# Safety assessment of microbial Polysaccharide Sanxan

**DOI:** 10.1371/journal.pone.0361184

**Published:** 2026-10-27

**Authors:** Yang Zhang, Haiqing Wu, Jiangyue Pi, Guizhi Quan, Qingyi Chuo, Xiaoyan Li, Haidong Huang

**Affiliations:** 1 Tianjin Key Laboratory of Intelligent Breeding of Major Crops, Tianjin, China; 2 Tianjin Agricultural University, Tianjin, China; 3 State Key Laboratory of Non-food Biomass Energy Technology, Nanning, China; Lorestan University, IRAN, ISLAMIC REPUBLIC OF

## Abstract

This study evaluated the oral safety of Sanxan to provide a basis for its application in food. The toxicological characteristics of Sanxan were assessed through an acute oral toxicity test, three genetic toxicity tests, a teratogenicity test in pregnant rats, and a 90-day oral toxicity test. The results showed that no obvious toxic symptoms or mortality were observed in the acute toxicity test, and the maximum tolerated dose was greater than 15 g/kg, indicating that Sanxan had no acute oral toxicity in the test animals. The results of three genetic toxicity tests-the Ames test, the micronucleus test, and the sperm abnormality test-were all negative, and the teratogenicity test in pregnant rats showed no maternal toxicity, embryotoxicity, or teratogenicity, indicating that Sanxan had no mutagenic or teratogenic effects under the tested conditions. The 90-day oral toxicity test showed that Sanxan at doses of 1.3, 2.6, and 5.2 g/kg body weight had no obvious adverse effects on body weight gain, total food intake, total food utilization, hematology, serum biochemistry, organ-to-body weight ratios, or pathological examination in the test animals, and the maximum no-observed-adverse-effect level (NOAEL) was 5.2 g/kg body weight. In summary, under the conditions of this study, Sanxan did not exhibit acute toxicity, genetic toxicity, teratogenicity, or subchronic toxicity, indicating that it has good safety under the tested conditions.

## 1 Introduction

Sanxan is a novel natural extracellular polysaccharide synthesized by *Sphingomonas sanxanigenens*, a bacterial strain belonging to the genus *Sphingomonas* [1]. It has an average molecular weight of 4.08 × 10^5^ Da and a polydispersity index of 1.07, the polysaccharide backbone is composed of four monosaccharides: D-mannose, D-glucuronic acid, L-rhamnose, and D-glucose, which form repeating units [2]. In 2020, the National Health Commission of China officially approved the application of Sanxan in the food industry as a thickener, stabilizer, and coagulant. Sanxan solution exhibits excellent gelation properties, emulsifying capacity, biocompatibility, and cost-effectiveness, garnering significant attention from researchers [3,4]. With its unique “cold-water gelation and heat-enhanced” characteristics, Sanxan demonstrates remarkable advantages in improving the quality of salt-free frozen foods. When added to frozen cooked noodles, it effectively inhibits texture deterioration during freeze-thaw cycles, enhancing hardness and chewiness while regulating water mobility and suppressing ice crystal growth to stabilize the gluten network [5–7]. In frozen dough systems, Sanxan reduces freezable water content, improving the specific volume and pore structure of steamed buns [8]. Furthermore, its interaction with proteins forms denser gel matrices with superior water-holding capacity and mechanical strength [9]. Studies reveal Sanxan’s multifaceted biomedical applications: As an exceptional suspending agent, it effectively loads insoluble usnic acid to create antibacterial dressings with sustained-release performance and wound-healing promotion [10]. pH-responsive Sanxan microcapsules protect medicines during gastrointestinal transit, offering new oral delivery strategies [11,12]. Leveraging its acid-induced gelation, emulsion-gel microcapsules achieve efficient protection and intestinal-targeted release of β-carotene [13]. However, most existing studies have mainly addressed its preparation and physicochemical or functional properties, while comprehensive toxicological evaluation remains scarce. Given its growing potential for use in foods, robust safety data are critically needed to ensure consumer health and regulatory acceptance [14,15]. This study comprehensively evaluates Sanxan’s toxicological profile under varying exposure conditions, to provide scientific evidence for evaluating the food safety and subsequent development/utilization of Sanxan.

## 2 Materials and methods

### 2.1 Sample

Food grade Sanxan (the fermentation product of *Sphingomonas sanxanigenens*) was obtained from Hebei Xinhe Biochemical Co. (Hebei, China). The molecular weight of Sanxan was 4.08 × 10^5^ Da and molecular mass dispersity value was 1.07 [2]. The maximum permitted levels of Sanxan are 1.3 g/kg in plant protein beverages, 1.4 g/kg in fruit/vegetable juice beverages, and 5.0 g/kg in meat sausages [16].

### 2.2 Animals and testing conditions

The clean-grade Sprague-Dawley (SD) rats and ICR mice used in the experiments were provided by Shanghai SLAC Laboratory Animal Co., Ltd. The experimental animal feed was provided by the Zhejiang Laboratory Animal Center, complying with the standard GB14924.1–2001. Prior to the start of the test, the animals were housed in an ecologically controlled environment in the animal facility, with a temperature of 20–23 ℃ and relative humidity of 50–70%. Except for the teratogenicity test in pregnant rats, which employed a 10-day acclimation period, all other experiments were conducted with a 3-day acclimation period. The Ames test was performed in a dedicated laboratory under sterile operating procedures, with a local cleanliness level of Class 100. Other conditions were the same as those in the ecologically controlled environment.

To ensure animal welfare, this study established clear humane endpoint criteria. During the experiment, animals were immediately euthanized if they exhibited any of the following conditions: body weight loss exceeding 20% of initial weight, inability to access food or water, persistent dyspnea, unresponsiveness to external stimuli, or signs of unrelieved severe pain or distress. Any animal meeting any of these criteria was immediately anesthetized with 100 mg/kg ketamine and euthanized by cervical dislocation to ensure rapid death, thereby minimizing suffering and guaranteeing humane treatment.

Throughout the experimental period (90 days), during which all animals were observed at least twice daily for general behavior, appearance, and physiological functions (including activity, grooming, feeding, and excretion) to assess health status, stress levels, and adverse reactions. Body weight was recorded weekly, along with monitoring of toxicological symptoms such as lethargy and dehydration. Throughout the experimental period, no animals died naturally prior to euthanasia. All remaining animals were euthanized upon completion of the study. All aspects in this study involving animal use and experimental procedure were performed in compliance with Technical Specifications for Health Food Inspection and Evaluation, published by China National Health Commission in 2003. To achieve a balance between ‘3R’ principle and data adequacy, we followed the National Institutes of Health guide for the care and use of Laboratory animals (NIH Publications No. 8023, revised 1978). All animal use and treatment have been approved by the Office of Laboratory Animal Welfare of Zhejiang Provincial Center for Disease Control and Prevention. The laboratory animal production license number is SCXK (Hu) 2012−0002, and the laboratory animal use license number is SYXK (Zhe) 2013−0190.

### 2.3 Methods

#### 2.3.1 Acute oral toxicity test and three genetic toxicity tests.

The experiments were conducted in accordance with the Technical Specifications for Testing and Evaluation of Health Foods (2003 Edition) issued by the Ministry of Health, performing the first and second stages of toxicity tests on Sanxan. This study was conducted in accordance with OECD Test Guidelines (OECD 408), and the animal experiments complied with China’s national food safety standards (GB 15193.3-5-2015, GB 15193.7−2015 and GB 15193.14−2015, 2015) [17].

**Acute oral toxicity test:** The experiment adopted the maximum tolerated dose (MTD) method. SD rats weighing 180–220 g (or clean-grade ICR mice weighing 18–22 g) were selected, with 10 animals of each sex. Sanxan was administered at a dose of 15 g/kg body weight as one dose group [18]. The sample was weighed and prepared with deionized water to the maximum gavage concentration of 25 g/100 mL. The solution was administered at 20 mL/kg body weight (0.2 mL/10 g for mice) per dose, with a 4-hour interval between doses, totaling three gavages. The animals were fasted overnight before the first gavage. After administration, the rats and mice were observed continuously for two weeks, and their general condition, toxic symptoms, and mortality were recorded.

**Ames test:** The experiment was conducted using four histidine-auxotrophic Salmonella typhimurium strains (TA97, TA98, TA100, and TA102), which had been biologically validated and were provided by the Department of Toxicology at the Shanghai Municipal Center for Disease Control and Prevention. The S9 (metabolic activation system) is the supernatant of liver homogenate obtained from SD rats induced by polychlorinated biphenyls (PCBs). Its biological activity was confirmed through testing with 2-aminofluorene (2-AF) and 1,8-dihydroxyanthraquinone. The 10% S9 mixture was prepared according to the Technical Specifications for Testing and Evaluation of Health Foods (2003). The plate incorporation method was used for the test: 1.0 g of Sanxan was dissolved in deionized water to a final volume of 20 mL, thoroughly mixed to prepare a solution of 50,000 µg/mL. This solution was then serially diluted five-fold with deionized water to obtain concentrations of 10,000 µg/mL, 2,000 µg/mL, 400 µg/mL, and 80 µg/mL. All concentrations were sterilized at 121 °C for 20 minutes. During the test, 0.1 mL of each concentration (equivalent to 5,000 µg/plate, 1,000 µg/plate, 200 µg/plate, 40 µg/plate, and 8 µg/plate) was added to each plate. A blank control group, solvent control group (deionized water), and positive control group were also included. For each strain and each test concentration, three parallel plates were prepared. The test was conducted both with and without the S9 metabolic activation system and repeated once. The number of revertant colonies on the medium was directly counted for each strain.

**Micronucleus test:** The experiment was conducted using a 24-hour interval oral gavage method. Ten ICR mice weighing 25–30 g, with an equal number of males and females, were selected. Sanxan was administered at three dose levels: 1.25 g/kg, 2.5 g/kg, and 5.0 g/kg body weight. A negative control group (deionized water) and a positive control group (cyclophosphamide at 60 mg/kg body weight) were also included. For the three dose groups, 6.5 g, 12.5 g, and 25 g of the sample were weighed and dissolved in deionized water to a final volume of 100 mL, preparing concentrations of 0.065 g/mL, 0.125 g/mL, and 0.25 g/mL, respectively. The solutions were administered by oral gavage at a dose of 20 mL/kg body weight, and the control groups were treated in the same manner. Six hours after the last administration of Sanxan, the animals were euthanized, and bone marrow was collected to prepare slides. The slides were fixed with methanol and stained with Giemsa. Under a microscope, 1,000 polychromatic erythrocytes (PCEs) were counted for each animal, and the number of PCEs containing micronuclei was recorded to calculate the micronucleus rate (‰). Additionally, 200 PCEs were counted to determine the number of normochromatic erythrocytes (NCEs) in the same field of view, and the PCE/RBC ratio was calculated.

**Sperm malformation test:** ICR mice weighing 25–35 g were selected, with five male mice in each group. Sanxan was administered at three dose levels: 1.25 g/kg, 2.5 g/kg, and 5.0 g/kg body weight. A negative control group (soybean oil) and a positive control group (mitomycin C at 2.0 mg/kg body weight) were also included. For the three dose groups, 6.5 g, 12.5 g, and 25 g of Sanxan were weighed and dissolved in deionized water to a final volume of 100 mL, preparing concentrations of 0.065 g/mL, 0.125 g/mL, and 0.25 g/mL, respectively. The solutions were administered by oral gavage at a dose of 20 mL/kg body weight for five consecutive days, and the control groups were treated in the same manner. On the 35th day after the first administration, the mice were euthanized by cervical dislocation. The epididymis was collected and placed in a dish containing an appropriate amount of physiological saline. It was then minced with ophthalmic scissors and filtered through four layers of lens paper. The filtrate was directly smeared onto slides, air-dried, fixed with methanol, and stained with 1% eosin. Under a microscope, 1,000 intact spermatozoa were observed for each mouse, and the types and numbers of abnormal spermatozoa were recorded to calculate the sperm abnormality rate (%).

### 2.4 Teratogenicity test in pregnant rats

Sanxan was thoroughly mixed with basal diet powder to prepare a premix for gavage administration. Female nulliparous rats weighing 230–320 g and male rats weighing 320–360 g were selected. Prior to the experiment, the animals were acclimatized to the animal room environment for 10 days. The experiment included three dose groups and a negative control group (basal diet). The low-, medium-, and high-dose levels were 1.3, 2.6, and 5.2 g/kg, respectively. Casein was supplemented to the high-dose premix to correct the protein content to 20%. After SD female and male rats were housed together overnight at a 1:1 ratio, vaginal plugs were checked every morning. The presence of a vaginal plug indicated successful mating, and that day was designated as “gestation day 0.” The identified pregnant rats were randomly assigned to groups, weighed, and numbered. From gestation days 7–16, the test substance was administered daily by oral gavage. Body weights were recorded on gestation days 0, 7, 12, 16, and 20, and the dosage was adjusted according to weight changes. On gestation day 20, the rats were euthanized by cervical dislocation. The uterus was removed, weighed, and examined to record the number of embryos, implantation sites, early resorptions, late deaths, and live fetuses, as well as the external morphology of the fetuses. The body weight, body length, and tail length of the fetuses were measured. Internal organ examination (focusing on the brain, heart, lungs, liver, stomach, intestines, kidneys, etc.) and skeletal examination (focusing on the skull, spine, sternum, ribs, and limb bones) were also performed.

### 2.5 90 d oral toxicity test

Eighty SD rats, with an equal number of males and females, were randomly divided into four groups: a negative control group (fed with basal diet) and low-, medium-, and high-dose groups (consistent with the teratogenicity test in pregnant rats). The test substance was uniformly mixed into the basal diet at 8% of the body weight and administered via feeding. The rats were housed individually, with free access to food and water, and observed continuously for 90 days. Body weight was recorded weekly, and food intake was recorded twice a week to calculate food utilization. Statistical analysis was performed using one-way analysis of variance (ANOVA), as described in Section 2.6. During the experiment (within the seventh week), blood was collected from the retro-orbital venous plexus. At the end of the experiment (the 13th week), the animals were fasted overnight after their final body weights were recorded. On the following day, after recording the fasting body weight, blood was collected from the jugular vein. Hematological parameters were measured using the Sysmex 1800i automated hematology analyzer. Blood biochemical parameters were measured using the TBA-40FR automated biochemistry analyzer. At the end of the experiment, all animals were subjected to gross necropsy. The liver, kidneys, spleen, and testes were weighed, and the organ-to-body weight ratios were calculated. Histopathological examinations were performed on the liver, kidneys, spleen, stomach, intestines, and testes (or ovaries). The tissues were fixed in 10% neutral formalin, embedded in paraffin, sectioned, and stained with hematoxylin and eosin (H&E) for light microscopic examination.

### 2.6 Statistical analysis

All data were analyzed using SPSS 11.5. In the acute oral toxicity test, all statistical analyses were performed separately by sex. In the three genetic toxicity tests, the teratogenicity test in pregnant rats, and the 90-day oral toxicity test, the homogeneity of variance was first tested: when the variance was homogeneous, one-way ANOVA was used for overall comparison, followed by pairwise comparison with the control group when a difference was found; when the variance was not homogeneous, the rank-sum test was used. In the three genetic toxicity tests and the teratogenicity test in pregnant rats, rates were analyzed by the chi-square (χ^2^) test. A value of P < 0.05 was considered statistically significant.

## 3 Results

### 3.1 Acute oral toxicity test and three genetic toxicity tests

#### 3.1.1 Acute oral toxicity test.

As shown in Table 1, the test animals received Sanxan by gavage in three portions, with a total dose of 15 g/kg body weight. During the 14-day observation period, no obvious toxic symptoms or mortality were observed. The acute oral maximum tolerated dose (MTD) for both male and female rats was greater than 15 g/kg body weight. These results indicate that Sanxan has no acute oral toxicity in the test animals.

**Table 1:** Results of the acute oral toxicity test of Sanxan.

Group	Sex	Dose (g/kg)	n	Initial Weight (g/$\overset{―}{x}\pm s$)	Final Weight (g/$\overset{―}{x}\pm s$)	Mortality (n)	Mortality rate (%)
**rat**	Male	15	10	203.0 ± 5.8	331.4 ± 10.1	0	0
	Female	15	10	204.5 ± 5.3	251.3 ± 8.3	0	0
**mouse**	Male	15	10	21.5 ± 0.5	36.9 ± 1.8	0	0
	Female	15	10	21.5 ± 0.3	28.6 ± 1.0	0	0

#### 3.1.2 Ames test.

For the four test strains (TA97, TA98, TA100, and TA102), the number of revertant colonies at different doses of Sanxan, both with and without the S9 metabolic activation, did not exceed twice that of the blank control group, and no obvious dose-response relationship was observed among the dose groups, as shown in Table 2.

**Table 2:** Ames test results of Sanxan ($\overset{―}{x}\pm s$).

Group	Dose (µg/plate)	Number of reverted colonies							
		TA97		TA98		TA100		TA102	
		-S9	+S9	-S9	+S9	-S9	+S9	-S9	+S9
Sanxan	8	126 ± 2.5	131 ± 2.6	31 ± 2.3	32 ± 1.5	130 ± 1.5	136 ± 2.6	258 ± 8.1	261 ± 4.7
	40	129 ± 1.2	136 ± 2.0	31 ± 1.5	33 ± 1.0	133 ± 3.2	137 ± 3.6	264 ± 4.9	271 ± 4.4
	200	132 ± 3.2	143 ± 6.0	33 ± 1.0	34 ± 1.2	137 ± 2.1	140 ± 3.2	275 ± 6.5	280 ± 4.9
	1000	137 ± 6.5	146 ± 7.0	36 ± 0.6	35 ± 1.2	140 ± 3.2	146 ± 2.0	281 ± 6.8	295 ± 6.6
	5000	141 ± 7.0	149 ± 8.5	37 ± 2.5	38 ± 3.0	144 ± 2.6	154 ± 4.4	286 ± 8.7	299 ± 10.8
Blank control	/	129 ± 5.5	131 ± 8.4	35 ± 1.5	37 ± 2.1	134 ± 3.5	142 ± 2.6	282 ± 5.6	284 ± 7.4
ICR-191	1.0	1116 ± 171.4	/	/	/	/	/	/	/
**Daunorubicin**	6.0	/	/	1511 ± 339.6	/	/	/	/	/
NaN3	1.5	/	/	/	/	1398 ± 193.3	/	/	/
**Mitomycin C**	0.5	/	/	/	/	/	/	1323 ± 185.9	/
2-AF	10	/	1446 ± 277.9	/	1367 ± 315.4	/	1197 ± 175.4	/	/
**1,8-Dihydroxyanthraquinone**	50	/	/	/	/	/	/	/	1377 ± 271.7

#### 3.1.3 Micronucleus test.

As shown in Table 3, the PCE/RBC ratio in each group was not less than 20% of that in the negative control group, indicating no evidence of bone marrow suppression. Statistical analysis (using the Poisson distribution) showed no significant differences in the micronucleus rates between any dose group and the negative control group (p > 0.05).

**Table 3:** Micronucleus test results of Sanxan on mouse bone marrow cells.

Sex	Group	n	Number of PCEs Examined	Number of PCEs with Micronuclei	Micronucleus Rate (‰)	P	PCE/RBC
Male	Negative Control	5	5000	6	1.20 ± 1.30	/	0.53 ± 0.05
	Low Dose	5	5000	6	1.20 ± 0.84	0.554	0.55 ± 0.02
	Medium Dose	5	5000	6	1.20 ± 1.10	0.554	0.55 ± 0.04
	High Dose	5	5000	7	1.40 ± 0.89	0.394	0.56 ± 0.06
	Positive Control	5	5000	138	27.60 ± 3.85	<0.001	0.51 ± 0.04
Female	Negative Control	5	5000	6	1.20 ± 0.84	/	0.61 ± 0.07
	Low Dose	5	5000	6	1.20 ± 1.30	0.554	0.60 ± 0.07
	Medium Dose	5	5000	5	1.00 ± 1.00	0.715	0.59 ± 0.06
	High Dose	5	5000	7	1.40 ± 1.14	0.394	0.61 ± 0.02
	Positive Control	5	5000	117	23.40 ± 2.88	<0.001	0.53 ± 0.03

#### 3.1.4 Sperm malformation test.

As shown in Table 4, compared with the negative control group (using the rank-sum test), there were no significant differences in the incidence of sperm abnormalities in mice treated with any dose of Sanxan (p > 0.05). In contrast, the positive control group (mitomycin C) showed a significantly increased rate of sperm abnormalities compared to the control group (p < 0.05). These results indicate that Sanxan has no toxic effects on the sperm of male test mice.

**Table 4:** Results of sperm malformation test of Sanxan on mice.

Group	n	Number of Sperm Examined	Number of Abnormal Sperm						Subtotal (n)	Berration rate (%)
			Hookless	Fat Head	Banana-Shaped	Double Head/ Tail	Folded	Amorphous		
**Negative Control**	5	5000	44	0	2	0	3	58	107	2.14 ± 0.57
**Low Dose**	5	5000	54	1	2	0	2	57	116	2.32 ± 0.52
**Medium Dose**	5	5000	54	0	3	0	2	50	109	2.18 ± 0.54
**High Dose**	5	5000	55	0	3	0	2	55	115	2.30 ± 0.42
**Positive Control**	5	5000	107	6	15	2	42	106	278	5.56 ± 0.94#

**Note: # indicates a significant difference compared to the negative control group, with P < 0.05.**

### 3.2 Teratogenicity test in pregnant rats

The study on the effects of Sanxan on the body weight of pregnant rats found no significant differences in body weight on gestation days 0, 7, 12, 16, and 20, as well as in total weight gain, between the negative control group and the dose groups (ANOVA, p > 0.05), as shown in Table 5. The study on its effects on rat fetuses (Tables 6, 7) found that the pregnancy rates in the negative control group and the low-, medium-, and high-dose groups were 86.7%, 93.3%, 93.3%, and 86.7%, respectively. The numbers of resorptions were 7, 6, 7, and 10, respectively, and the numbers of dead fetuses were all 0. Furthermore, there were no significant differences in uterine weight with fetuses, average number of fetuses, average fetal weight, or average fetal body length between the low-, medium-, and high-dose groups and the negative control group (ANOVA, p > 0.05). In the study on the effects of Sanxan on fetal skeletal development (Table 8), there were no significant differences in the number of fetal sternal bones (including 6, 5, and 4 pieces) or the number of fifth sternal bones appearing as dots between the low-, medium-, and high-dose groups and the negative control group (chi-square test, p > 0.05). No significant differences were observed in the number of fetal ribs (13 pairs or 12 pairs) between the experimental groups and the negative control group. The numbers of fetuses with incomplete ossification of the parietal bone were 1, 0, 1, and 0, respectively. In the study on the effects of the sample on fetal visceral development (Table 9), individual fetuses in the negative control group and the low-, medium-, and high-dose groups showed hydronephrosis and renal pelvis dilation, with the numbers of affected fetuses being 2, 2, 1, and 1, respectively. However, no abnormalities were found in the brain, heart, lungs, liver, stomach, intestines, testes (uterus), or bladder in any of the examined fetuses. In conclusion, no significant maternal toxicity, embryotoxicity, or teratogenicity was observed in any of the dose groups.

**Table 5:** Effects of Sanxan on the body weight of pregnant rats ($\overset{―}{x}\pm s$).

Group	n	Gross weight gain	Gestation days				
			0 (d)	7 (d)	12 (d)	16 (d)	20 (d)
**Negative Control**	12	176.2 ± 21.7	268.1 ± 15.3	307.6 ± 17.4	337.3 ± 17.8	365.0 ± 19.6	444.3 ± 28.2
**Low Dose**	12	166.2 ± 33.4	272.9 ± 20.2	312.0 ± 21.4	342.9 ± 25.5	371.5 ± 31.1	439.1 ± 41.0
**Medium Dose**	12	182.7 ± 15.0	281.3 ± 16.7	321.7 ± 18.2	346.6 ± 20.8	371.5 ± 24.8	463.9 ± 23.5
**High Dose**	12	182.5 ± 35.7	274.9 ± 17.4	320.7 ± 28.7	340.8 ± 37.7	380.2 ± 45.3	457.4 ± 45.8
F P		1.074	1.315	1.311	1.155 (H)	0.509	1.417
		0.369	0.280	0.281	0.764	0.678	0.249

**Note: (H) for Kruskal-Wallis test**

**Table 6:** Effects of Sanxan on rat fetuses (Part 1).

Group	Fertilized rat (n)	Pregnant rats (n)	Pregnancy rate (%)	Resorbed Fetuses (n)	Dead Fetuses (n)
**Negative Control**	15	13	86.7	7	0
**Low Dose**	15	14	93.3	6	0
**Medium Dose**	15	14	93.3	7	0
**High Dose**	15	13	86.7	10	0

**Table 7:** Effects of Sanxan on rat fetuses (Part 2).

Group	Number of fetuses (n)	Mean Number of Fetuses (n)	Uterine Weight with Fetuses (g)	Mean Fetal Weight (g)	Mean Fetal Body Length (cm)
**Negative Control**	193	14.8 ± 2.3	98.2 ± 15.9	4.26 ± 0.61	3.85 ± 0.19
**Low Dose**	208	14.9 ± 4.3	98.2 ± 25.9	4.35 ± 0.68	3.86 ± 0.24
**Medium Dose**	225	16.1 ± 2.1	103.6 ± 12.9	4.18 ± 0.48	3.80 ± 0.15
**High Dose**	206	15.9 ± 1.2	104.4 ± 14.9	4.31 ± 0.67	3.82 ± 0.19
H P		0.742	0.458	0.181	0.223
		0.532	0.713	0.909	0.880

**Table 8:** Effects of Sanxan on fetal skeletal development.

Group	Number of Cases (n)	Ribs		Sternal Bones				Incomplete Ossification of the Parietal Bone (n)
		13 pairs	12 pairs	6 Piece (%)	5 Piece (%)	4 Piece (%)	The Fifth is Dot-Like (%)	
**Negative Control**	99	99	0	95 (96.0)	4 (4.0)	0 (0)	5 (5.1)	1
**Low Dose**	108	107	1	102 (94.4)	6 (5.6)	0 (0)	8 (7.4)	0
**Medium Dose**	116	115	1	106 (91.4)	7 (6.0)	3 (2.6)	5 (4.3)	1
**High Dose**	106	105	1	96 (90.6)	7 (6.6)	3 (2.8)	8 (7.5)	0
X^2^				3.114	0.700		1.557	
p				0.429	0.429		0.429	

**Table 9:** Effects of Sanxan on fetal visceral development.

Group	Number of Fetuses Examined (n)	Number of Fetuses with No Abnormalities (n) (%)	Number of Fetuses with Abnormalities (Hydronephrosis and Renal Pelvis Dilation) (n) (%)
**Negative Control**	94	92 (97.9)	2
**Low Dose**	100	98 (98.0)	2
**Medium Dose**	109	108 (99.1)	1
**High Dose**	100	99 (99.0)	1

### 3.3 90 d oral toxicity test

In the early stages of the experiment, some animals in the medium- and high-dose groups exhibited sticky stool with a small amount of mucus, which gradually disappeared by weeks 3–4, and the stool characteristics of all animals returned to normal. This transient phenomenon can be attributed to the well-documented effects of high dietary fiber intake. Supplementation with soluble dietary fiber increases fecal water content, leading to sticky stool [19]. No other abnormal symptoms or signs were observed in the animals during the remainder of the experiment, and no animal deaths occurred.

#### 3.3.1 Effects of Sanxan on body weight gain, total food intake, and total food utilization rate in rats.

As shown in Table 10, the total food utilization rate in the low-dose group of female rats was higher than that in the negative control group, with a statistically significant difference (p < 0.05). For all other dose groups, there were no significant differences in weekly food utilization rates compared with the negative control group (p > 0.05). Similarly, no significant differences were observed in weekly body weight, food intake, body weight gain, total food intake, or total food utilization rate between any dose group and the negative control group (p > 0.05), except for the aforementioned increase in total food utilization rate in the low-dose female group. This difference was observed only in the low-dose group, showed no dose-response relationship, and an increased food utilization rate is not considered an adverse effect. Combined with the absence of significant changes in body weight, food intake, and weight gain, these findings indicate that Sanxan had no obvious adverse effects on body weight gain, food intake, or food utilization rate in rats.

**Table 10:** Changes in total food utilization rate in rats during the 90-Day oral toxicity test of Sanxan.

Sex	Group	n	Body Weight Gain (g)	Total Food Intake (g)	Total Food Utilization Rate (%)
Male	**Negative Control**	10	465.3 ± 28.2	3236.1 ± 145.4	14.4 ± 0.5
	**Low Dose**	10	430.7 ± 51.3	3039.8 ± 207.4	14.1 ± 0.9
	**Medium Dose**	10	442.7 ± 23.9	3136.5 ± 132.9	14.1 ± 0.5
	**High Dose**	10	435.8 ± 45.8	3179.1 ± 227.3	13.7 ± 1.1
	F P		1.528	2.055	3.552 (H)
			0.224	0.123	0.314
Female	**Negative Control**	10	223.6 ± 24.2	2192.4 ± 195.4	10.2 ± 0.4
	**Low Dose**	10	231.3 ± 16.7	2127.2 ± 162.5	10.9 ± 0.5#
	**Medium Dose**	10	205.4 ± 25.7	2026.3 ± 164.5	10.1 ± 0.7
	**High Dose**	10	216.7 ± 24.7	2149.0 ± 167.5	10.1 ± 0.7
	F P		2.267	1.655	4.092
			0.097	0.194	0.013

**Note: (H) for Kruskal-Wallis Test; # indicates a significant difference compared to the negative control group (p < 0.05)**

#### 3.3.2 Effects of Sanxan on hematological parameters in rats.

As shown in Table 11, there were no significant differences in hemoglobin levels, red blood cell counts, or white blood cell counts between any dose group and the negative control group at both the mid-term and final stages of the experiment (p > 0.05). Additionally, all hematological parameters in the dose groups showed no statistically significant differences compared to the negative control group (p > 0.05). The results indicate that all hematological parameters remained within the normal range during the 90-day oral toxicity test of Sanxan, and Sanxan had no adverse effects on hematological indicators.

**Table 11:** Hematological parameters in the 90-Day oral toxicity test of Sanxan ($\overset{―}{x}\pm s$).

Sex	Group	n	Mid-Term			Final-Term		
			Hemoglobin (g/L)	Red Blood Cell (x10^12^/L)	White Blood Cell (x10^9^/L)	Hemoglobin (g/L)	Red Blood Cell (x10^12^/L)	White Blood Cell (x10^9^/L)
Male	Negative Control	10	167.3±21.6	8.7±1.2	11.2±5.5	160.3±5.5	8.8±0.2	10.9±3.2
	Low Dose	10	171.2±21.6	9.2±1.2	9.4±3.4	158.5±4.3	8.8±0.3	11.2±3.9
	Medium Dose	10	184.2±7.9	10.0±0.7	9.9±5.0	156.2±5.7	8.7±0.3	8.7±1.6
	High Dose	10	179.0±15.6	9.4±0.7	8.9±3.3	162.4±6.9	8.9±0.3	8.4±1.5
	F		1.870	2.823	0.482	2.163	1.008	4.594 (H)
	P		0.152	0.052	0.697	0.109	0.400	0.204
Female	Negative Control	10	174.2±7.6	9.3±0.4	8.9±2.4	158.4±6.0	8.7±0.3	7.1±1.6
	Low Dose	10	167.6±4.9	9.0±0.4	7.8±2.8	153.2±4.7	8.4±0.3	5.9±1.4
	Medium Dose	10	164.2±18.2	8.9±1.1	8.6±2.2	157.5±4.3	8.7±0.3	7.8±1.5
	High Dose	10	168.0±11.9	9.1±0.6	9.1±3.1	158.6±5.1	8.7±0.2	7.2±1.7
	F		1.250	0.603	0.469	2.468	2.824	2.734
	P		0.306	0.617	0.706	0.078	0.052	0.058
**Leukocyte differential count**								
**Sex**	**Group**	**n**	**Lymphocytes** **(%)**	**Monocytes** **(%)**	**Neutrophils** **(%)**	**Eosinophils** **(%)**	**Basophils** **(%)**	
Male	Negative Control	10	67.63±2.66	3.13±0.73	26.66±2.06	2.49±0.65	0~0.3	
	Low Dose	10	66.75±2.15	3.14±0.80	27.51±2.48	2.53±0.81	0~0.3	
	Medium Dose	10	66.86±3.46	2.93±0.82	27.85±3.19	2.33±0.46	0~0.1	
	High Dose	10	65.37±3.49	3.02±0.55	29.36±3.29	2.17±0.36	0~0.2	
	F		0.987	0.185	1.618	0.763		
	P		0.410	0.906	0.202	0.522		
Female	Negative Control	10	69.31±2.04	2.33±0.36	25.35±1.96	2.97±1.24	0~0.2	
	Low Dose	10	68.23±3.65	2.40±0.61	26.56±3.30	2.77±0.96	0~0.2	
	Medium Dose	10	68.36±3.30	2.69±0.84	26.38±1.91	2.55±0.80	0~0.1	
	High Dose	10	66.88±3.41	2.62±0.65	27.45±2.31	3.03±1.00	0~0.2	
	F		0.999	0.730	1.249	0.461		
	P		0.404	0.541	0.306	0.711		

**Note: (H) for Kruskal-Wallis Test**

#### 3.3.3 Effects of Sanxan on blood biochemical parameters in rats.

As shown in Table 12, there were no significant differences in serum alanine aminotransferase (ALT), aspartate aminotransferase (AST), blood urea nitrogen (BUN), creatinine, cholesterol, triglycerides, blood glucose, total protein, albumin, globulin, or albumin-to-globulin ratio between any dose group and the negative control group during the experiment (p > 0.05). The results indicate that all blood biochemical parameters remained within the normal range during the 90-day oral toxicity test of Sanxan, and Sanxan had no adverse effects on blood biochemical indicators.

**Table 12:** Blood biochemical parameters in the 90-Day oral toxicity test of Sanxan.

Sex	Group	n	Alanine Aminotransferase (U/L)	Aspartate Aminotransferase (U/L)	Blood Urea Nitrogen (mmol/L)	Creatinine (umol/L)	Triglycerides (mmol/L)	Cholesterol (mmol/L)
Male	Negative Control	10	53.6±9.6	278.9±55.4	6.0±0.7	67.5±3.7	0.72±0.20	1.81±0.19
	Low Dose	10	57.7±8.2	293.3±47.5	5.6±0.4	68.6±3.9	0.89±0.18	1.81±0.33
	Medium Dose	10	57.5±7.0	294.2±43.9	6.0±0.8	71.7±5.5	0.80±0.21	1.59±0.20
	High Dose	10	62.5±11.4	309.1±64.9	6.5±0.9	68.6±3.3	0.67±0.16	1.69±0.30
	F		1.565	0.531	2.371	1.866	2.819	1.616
	P		0.215	0.664	0.087	0.153	0.053	0.203
Female	Negative Control	10	46.9±9.6	242.3±57.3	6.9±1.1	79.1±3.4	0.54±0.12	2.21±0.30
	Low Dose	10	55.7±8.3	306.8±73.0	6.7±0.8	79.8±3.4	0.67±0.31	2.24±0.41
	Medium Dose	10	52.6±8.7	281.9±21.5	6.3±0.4	77.3±3.6	0.56±0.21	2.16±0.36
	High Dose	10	55.9±10.5	309.5±67.8	5.9±0.6	75.5±4.0	0.61±0.23	2.32±0.50
	F		2.040	2.839	7.741 (H)	2.833	0.683	0.281
	P		0.126	0.052	0.052	0.052	0.568	0.838
**Sex**	**Group**	**n**	**Blood Glucose** **(mmol/L)**	**Total Protein** **(g/L)**	**Albumin** **(g/L)**	**Globulin** **(g/L)**	**Albumin-to-Globulin Ratio**	
Male	Negative Control	10	5.52±0.50	61.0±1.3	37.2±1.0	23.9±0.6	1.56±0.05	
	Low Dose	10	5.47±0.80	61.6±1.8	37.5±0.6	24.0±1.6	1.57±0.11	
	Medium Dose	10	5.58±0.90	62.0±3.2	37.9±1.2	24.1±2.2	1.58±0.11	
	High Dose	10	4.73±0.77	61.5±2.6	37.6±1.1	23.9±2.1	1.58±0.13	
	F		2.821	0.269	0.797	0.159 (H)	0.117	
	P		0.053	0.848	0.504	0.984	0.950	
Female	Negative Control	10	5.93±1.29	70.0±3.9	42.2±2.1	27.8±2.1	1.52±0.08	
	Low Dose	10	4.91±0.57	69.2±4.5	42.3±2.7	26.9±2.0	1.57±0.07	
	Medium Dose	10	5.21±0.60	69.0±2.3	41.6±1.5	27.4±1.5	1.52±0.09	
	High Dose	10	5.17±0.58	66.6±3.7	41.0±2.3	25.6±1.9	1.61±0.11	
	F		2.847	1.601	0.717	2.645	2.312	
	P		0.051	0.206	0.549	0.064	0.093	

**Note: (H) for Kruskal-Wallis Test**

#### 3.3.4 Effects of Sanxan on organ-to-body weight ratios in rats.

As shown in Table 13, there were no significant differences in the absolute weights of the liver, spleen, kidneys, and testes, or in the organ-to-body weight ratios between any dose group and the negative control group (p > 0.05). These results indicate that the organ weights and organ-to-body weight ratios remained within normal ranges during the 90-day oral toxicity test of Sanxan.

**Table 13:** Effects of Sanxan on organ-to-body weight ratios in rats in the 90-Day oral toxicity test.

Sex	Group	n	Fasting Body Weight (g)	Liver-to-Body Weight Ratio (%)	Kidney-to-Body Weight Ratio (%)	Spleen-to-Body Weight Ratio (%)	Testis-to-Body Weight Ratio (%)
Male	**Negative Control**	10	512.8 ± 35.3	2.54 ± 0.27	0.72 ± 0.03	0.18 ± 0.03	0.77 ± 0.08
	**Low Dose**	10	475.4 ± 45.3	2.54 ± 0.16	0.70 ± 0.04	0.17 ± 0.03	0.82 ± 0.08
	**Medium Dose**	10	482.0 ± 22.1	2.49 ± 0.14	0.71 ± 0.05	0.16 ± 0.02	0.79 ± 0.06
	**High Dose**	10	468.3 ± 42.7	2.41 ± 0.09	0.72 ± 0.05	0.17 ± 0.03	0.84 ± 0.08
	F P		2.736	1.156	0.442	0.874	1.753
			0.058	0.340	0.725	0.463	0.174
Female	**Negative Control**	10	289.1 ± 22.6	2.54 ± 0.15	0.69 ± 0.05	0.19 ± 0.02	/
	**Low Dose**	10	284.7 ± 18.7	2.50 ± 0.24	0.68 ± 0.05	0.20 ± 0.03	/
	**Medium Dose**	10	264.6 ± 22.3	2.48 ± 0.09	0.67 ± 0.03	0.19 ± 0.02	/
	**High Dose**	10	271.0 ± 23.2	2.46 ± 0.10	0.71 ± 0.05	0.20 ± 0.03	/
	F P		2.779	2.679 (H)	1.205	0.419	/
			0.055	0.444	0.322	0.741	/

**Note: (H) for Kruskal-Wallis Test**

#### 3.3.5 Pathological examination of Sanxan.

At the end of the experiment, gross examinations were performed on the liver, kidneys, spleen, gastrointestinal tract, testes, and ovaries of all experimental rats (Table 14). A total of 80 rats were dissected, and no significant abnormalities were observed during gross dissection. Upon detailed microscopic examination, mild liver lesions were found in some rats in both the negative control group and the high-dose group. These lesions included small focal inflammatory cell infiltration in the liver lobules, mild inflammatory cell infiltration in the portal areas, and focal or small focal hepatocyte necrosis accompanied by inflammatory cell infiltration. In a few rats, a tiny granuloma was observed in the liver parenchyma. In the kidneys, a small number of rats exhibited granular degeneration of renal tubular epithelial cells, mild inflammatory cell infiltration in the renal cortical interstitium, and focal glomerular calcification, although the overall severity of these lesions was mild. Additionally, mild sinusoidal dilation and congestion were observed in the spleen of a few rats in the negative control group. The aforementioned histological changes were sporadic and showed no significant differences between the negative control group and the high-dose group. The above histological changes occurred sporadically as individual cases and were also observed in the negative control group. The number of lesions did not increase with increasing dose of the test substance, and no dose-response relationship was observed, consistent with the characteristics of background lesions in laboratory animals. No toxic pathological damage induced by the test substance was observed. Due to the low incidence of these lesions, no statistical comparison of their incidence rates between groups was performed in this study, and their biological significance remains to be further verified with larger sample sizes and more in-depth investigations. Taken together, the results of all parameters indicate that no obvious toxic reactions were observed in the 90-day feeding study of Sanxan in rats, and the no-observed-adverse-effect level (NOAEL) was determined to be 5.2 g/kg body weight (high-dose group). This value is of a similar order of magnitude to the NOAELs reported for other widely used microbial polysaccharide food additives, such as gellan gum and xanthan gum, further demonstrating that Sanxan exhibits good tolerability under the conditions of this study [20–23].

**Table 14:** Results of histopathological examination.

Organs	Pathological Changes (Cases)	Negative Control		High Dose	
		Male (n = 10)	Female (n = 10)	Male (n = 10)	Female (n = 10)
Liver	Mild Congestion of Central Veins in Lobules	0	0	0	0
	Sinusoidal Congestion in the Liver	0	0	0	0
	Granular Degeneration of Cells	0	0	0	0
	**Vacuolar Degeneration of Cells**	0	0	0	0
	Small Focal Inflammatory Cell Infiltration in Lobules	1	1	1	1
	Inflammatory Cell Infiltration in Portal Areas	2	0	0	0
	Focal Necrosis with Inflammatory Cell Infiltration	0	2	2	0
	Microgranuloma	1	0	0	0
**Kidneys**	Swelling of Renal Tubular Epithelial Cells	1	0	1	0
	Patchy Inflammatory Cell Infiltration in the Cortical Interstitium	0	0	1	0
	Mild Calcification of Glomeruli	0	0	1	0
	Mild Dilation and Congestion of Cortical Interstitial Blood Vessels	0	0	0	0
	Calcium Salt Deposition in Renal Tubules	0	0	0	0
	Hydropic Degeneration of Papillary Duct Epithelial Cells	0	0	0	0
**Spleen**	Mild Dilation and Congestion of Sinusoids	0	1	0	0
	Thickening of the Lymphoid Sheath Around Central Arteries	0	0	0	0
	Reactive Hyperplasia of Lymphoid Follicles	0	0	0	0
Gastrointestinal Tract	Congestion of Microvessels in the Lamina Propria	0	0	0	0
	Congestion of Blood Vessels in the Submucosa	0	0	0	0
	Degeneration and Necrosis of the Mucosal Epithelium	0	0	0	0
	Inflammatory Cell Infiltration in the Mucosal Layer	0	0	0	0
**Testes**	Reduction in Germ Cells and Spermatogenic Dysfunction	0	0	0	0
	Atrophy of Seminiferous Tubules	0	0	0	0
	Hyperplasia of Interstitial Cells	0	0	0	0
Ovaries	Ovarian Cysts	0	0	0	0
	Ovarian Hemorrhage	0	0	0	0
	Hyperplasia of Interstitial Cells	0	0	0	0

## 4 Conclusion

To evaluate the oral safety of Sanxan, this study conducted a toxicological assessment. Under the conditions of this study, no obvious toxic symptoms or mortality were observed in the acute toxicity test, and no Sanxan-related acute oral toxicity was observed. Genetic toxicity tests (including Ames test, micronucleus test, and sperm abnormality test) all showed negative results, demonstrating no damage to animal somatic or germ cells and no detectable genotoxicity. Teratogenicity studies confirmed Sanxan caused no toxic effects in pregnant rats or their fetuses, with no observed teratogenic potential. The 90-day oral toxicity test revealed no significant toxic effects, establishing the no-observed-adverse-effect level (NOAEL) at 5.2 g/kg body weight. Framing Sanxan’s NOAEL within this established range indicates a comparable safety profile and suggests that Sanxan does not pose a health risk under the intended conditions of use. These results demonstrate the favorable safety profile of Sanxan in animal studies, providing a scientific basis for its further development and rational utilization.

## Supporting information

S1 File Raw data and test reports of Sanxan, including acute oral toxicity tests in rats and mice, the Ames test, the mouse micronucleus test, the mouse sperm abnormality test, the teratogenicity test, and the 90-day oral toxicity test. (PDF)
