# Supplementary material for: Safety assessment of microbial Polysaccharide Sanxan
Source: PLoS One. 2026 Oct 27;21(10):e0361184. doi: 10.1371/journal.pone.0361184 (PMC13712527; doi:10.1371/journal.pone.0361184)
Supplement: Supplementary file 1 [file pone.0361184.s001.pdf]

The test sample of Sanxan is an off-white powder. The submitting party provided a maximum recommended human dietary intake of 1.3 g/person/day. According to the requirements of the Technical Standards for Testing and Assessment of Health Food (2003 Edition) issued by the Ministry of Health, the first and second stage toxicity tests were conducted on this sample.

Experimental results: The acute oral maximum tolerated dose in both male and female rats and mice was greater than 15 g/kg body weight, belonging to the non-toxic grade; the Ames test, mouse bone marrow cell micronucleus test, and mouse sperm abnormality test results were negative. This indicates that under the conditions of this experiment, the sample did not show mutagenicity.

The remainder of this page is blank.

Rat acute oral toxicity test, mouse acute oral toxicity test, Ames test, mouse micronucleus test, mouse sperm abnormality test

1.Sample: The recommended human intake of Sanxan is 1.3 g/60 kg/day, production date 2014.04.21, batch number 1404021. For all rat and mouse experiments and the Ames test, deionized water was used as the solvent to prepare different concentrations for testing. The maximum gavage concentration prepared for animal experiments was 25 g/100 mL.

2.Experimental animals and testing conditions:

Clean-grade SD rats and ICR mice were both provided by Shanghai Slac Laboratory Animal Co., Ltd. (Laboratory Animal Production License No. SCXK (Hu) 2012-0002). Laboratory animal feed was provided by Zhejiang Laboratory Animal Center, implementing standard GB14924.1-2001.

Testing environmental conditions: barrier environment, temperature range 20–23°C, relative humidity 50–70%. Laboratory Animal Use License No. SYXK (Zhe) 2013-0190. The experimental animals were acclimated in the animal room environment for 3 days before testing. The Ames test was conducted in a dedicated laboratory according to aseptic operating procedures. The laboratory cleanliness was local class 100, temperature range 20–23°C, relative humidity range 50–70%.

3. Experimental methods:

3.1 Rat acute oral toxicity test:

3.1.1 Experimental animals: SD rats, body weight 180–220 g.

3.1.2 Dosage and administration method: The maximum tolerated dose method was used in the experiment. 20 animals, half male and half female, fasted overnight before exposure. One dose group of sample 15 g/kg body weight was set. The test substance was administered by oral gavage. The sample was prepared with deionized water to the maximum gavage concentration of 25 g/100 mL. At 20 mL/kg body weight each time, with an interval of 4 h, gavage was performed in 3 divided doses.

3.1.3 Observation indicators: After exposure, the general condition, toxic symptoms, and death of rats were observed. The observation period was two weeks.

3.2 Mouse acute oral toxicity test:

3.2.1 Experimental animals: ICR mice, clean grade, body weight 18–22 g.

3.2.2 Dosage and administration method: The maximum tolerated dose method was used in the experiment. 20 animals, half male and half female, fasted overnight before exposure. One dose group of sample 15 g/kg body weight was set. The test substance was administered by oral gavage. The sample was prepared with deionized water to the maximum gavage concentration of 25 g/100 mL. At 0.2 mL/10 g body weight each time, with an interval of 4 h, gavage was performed in 3 divided doses.

3.2.3 Observation indicators: After exposure, the general condition, toxic symptoms, and death of mice were observed. The observation period was two weeks.

3.3 Ames test:

3.3.1 Test strains: Histidine auxotrophic Salmonella typhimurium TA97, TA98, TA100, and TA102 were selected, provided by the Toxicology Department of Shanghai Municipal Center for Disease Control and Prevention, and preserved in our laboratory. The test was conducted after biological identification was qualified.

3.3.2 Metabolic activation system (S9): S9 is the liver homogenate supernatant obtained from polychlorinated biphenyl-induced SD rats. It was tested with 2-AF and 1,8-dihydroxyanthraquinone and had biological activity. The 10% S9 mixture was prepared according to the formula recommended in the Technical Standards for Testing and Assessment of Health Food (2003).

3.3.3 Dose selection: The plate incorporation method was used in the experiment. The highest dose selected was 5000 μg/plate. Weigh 1.0 g of sample and add deionized water to 20 mL, mix thoroughly, prepare to 50000 μg/mL, and serially dilute 5-fold with deionized water to equivalent concentrations of 10000, 2000, 400, and 80 μg/mL. All concentrations were sterilized by moist heat at 121°C for 20 min before use in the test. During the test, 0.1 mL was added to each culture dish, corresponding to doses of 5000, 1000, 200, 40, and 8 μg/plate. Blank control group, solvent control group (deionized water), and positive control group were also set. Three parallel plates were set for each strain at each test concentration, and the test was conducted under conditions with and without S9. The test was repeated once.

3.3.4 Observation indicators: Directly count the number of revertant colonies of each strain on the culture medium.

3.4 Mouse bone marrow cell micronucleus test:
3.4.1 Experimental animals: ICR mice, 25–30 g.
3.4.2 Dosage and administration method: Three dose groups of sample 1.25, 2.5, and 5.0 g/kg body weight were set (equivalent to 58, 115, and 231 times the recommended human dietary intake, respectively), and a negative control group (deionized water) and a positive control group (cyclophosphamide 60 mg/kg body weight) were also set. Each group had 10 mice, half male and half female. The sample was administered twice with an interval of 24 h. For the three dose groups, 6.5, 12.5, and 25 g of sample were respectively weighed and added with deionized water to 100 mL to prepare 0.065, 0.125, and 0.25 g/mL, and gavaged at 20 mL/kg body weight. The control groups were treated the same way. Animals were sacrificed 6 h after the last administration of the sample. Bone marrow was taken for smear preparation, fixed with methanol, and stained with Giemsa.
3.4.3 Observation indicators: Under the microscope, 1000 polychromatic erythrocytes (PCE) were counted for each animal, and the number of PCE containing micronuclei was observed, and the micronucleus rate (%) was calculated. The number of NCE in 200 PCE fields was also counted, and the PCE/RBC ratio was calculated.
3.5 Mouse sperm abnormality test:
3.5.1 Experimental animals: ICR mice, body weight 25–35 g.
3.5.2 Dosage and administration method: Three dose groups of sample 1.25, 2.5, and 5.0 g/kg body weight were set (equivalent to 58, 115, and 231 times the recommended human dietary intake, respectively), and a negative control group (soybean oil) and a positive control group (mitomycin C 2.0 mg/kg body weight) were also set. Each group had 5 male mice. For the three dose groups, 6.5, 12.5, and 25 g of sample were respectively weighed and added with deionized water to 100 mL to prepare 0.065, 0.125, and 0.25 g/mL, and gavaged at 20 mL/kg body weight. Administration continued for five consecutive days. The control groups were treated the same way. On the 35th day after the first administration of the sample, the mice were sacrificed by cervical dislocation. The epididymis was taken, placed in a petri dish containing an appropriate amount of physiological saline, cut into pieces with ophthalmic scissors, filtered through four layers of lens paper, and directly smeared. Air-dried naturally, fixed with methanol, and stained with 1% eosin.
3.5.3 Observation indicators: Under the microscope, 1000 complete sperm were observed for each mouse, the types and numbers of abnormal sperm were recorded, and the sperm abnormality rate (%) was calculated.
4 Experimental results:
4.1 Rat acute oral toxicity test:
The results are shown in Table 1. After exposure, no abnormal manifestations were observed in the experimental rats. The acute oral maximum tolerated dose in both male and female sexes was greater than 15 g/kg body weight.

Table 1. Results of the sample in the rat acute oral toxicity test

Sex	Dose g/kg	Number of animals	Initial weight g, $\bar{x} \pm s$	Final weight g, $\bar{x} \pm s$	Number of dead animals	Mortality %
Female	15	10	204.5±5.3	251.3±8.3	0	0
Male	15	10	203.0±5.8	331.4±10.1	0	0

4.2 Mouse acute oral toxicity test:
The results are shown in Table 2. After exposure, no abnormal manifestations were observed in the experimental mice. The acute oral maximum tolerated dose in both male and female sexes was greater than 15 g/kg body weight.

Table 2. Results of the sample in the mouse acute oral toxicity test

Sex	Dose g/kg	Number of animals	Initial weight g, $\bar{x} \pm s$	Final weight g, $\bar{x} \pm s$	Number of dead animals	Mortality %
Female	15	10	21.5±0.3	28.6±1.0	0	0
Male	15	10	21.5±0.5	36.9±1.8	0	0

4.3 Ames test:
The experimental results are shown in Tables 3 and 4. The number of revertant colonies of the sample at different doses under conditions with and without S9 did not exceed twice the number of revertant colonies of the blank control group and solvent control group, and there was no obvious dose-response relationship among the dose groups.

Table 3. Ames test results of the test substance (first time) ($\bar{x} \pm S$)

Group	Dose (μg/mL)	Number of reverted colonies							
		TA97		TA98		TA100		TA102	
		-S9	+S9	-S9	+S9	-S9	+S9	-S9	+S9
Sanxan	8	126±2.5	131±2.6	31±2.3	32±1.5	130±1.5	136±2.6	258±8.1	261±4.7
	40	129±1.2	136±2.0	31±1.5	33±1.0	133±3.2	137±3.6	264±4.9	271±4.4
	200	132±3.2	143±6.0	33±1.0	34±1.2	137±2.1	140±3.2	275±6.5	280±4.9
	1000	137±6.5	146±7.0	36±0.6	35±1.2	140±3.2	146±2.0	281±6.8	295±6.6
	5000	141±7.0	149±8.5	37±2.5	38±3.0	144±2.6	154±4.4	286±8.7	299±10.8
Blank control	/	129±5.5	131±8.4	35±1.5	37±2.1	134±3.5	142±2.6	282±5.6	284±7.4
Solvent control	DMSO	127±6.8	132±7.0	36±1.0	38±3.2	136±4.0	143±3.8	280±11.0	283±14.7
ICR-191	1.0	1116±171.4	/	/	/	/	/	/	/
Daunorubicin	6.0	/	/	1511±339.6	/	/	/	/	/
NaN3	1.5	/	/	/	/	1398±193.3	/	/	/
Mitomycin C	0.5	/	/	/	/	/	/	1323±185.9	/
2-AF	10	/	1446±277.9	/	1367±315.4	/	1197±175.4	/	/
1,8-Dihydroxyanthraquinone	50	/	/	/	/	/	/	/	1377±271.7

Table 4. Ames test results of the test substance (second time) ($\bar{x} \pm S$)

Group	Dose (μg/mL)	Number of reverted colonies							
		TA97		TA98		TA100		TA102	
		-S9	+S9	-S9	+S9	-S9	+S9	-S9	+S9
Sanxan	8	126±4.0	129±3.8	31±1.2	33±2.3	129±4.0	130±1.5	251±6.4	264±5.5
	40	129±5.0	132±5.6	33±2.9	33±1.5	133±3.6	136±1.5	253±3.8	267±3.2
	200	131±4.4	138±2.9	33±0.6	35±1.2	136±2.0	138±2.1	264±8.1	280±3.0
	1000	134±1.5	139±6.6	35±1.0	37±1.5	144±5.0	145±4.2	275±6.1	290±5.3
	5000	140±2.1	144±4.6	36±2.3	39±2.6	143±3.5	149±7.4	289±7.0	299±1.5
Blank control	/	129±4.5	139±6.1	36±2.0	37±2.1	131±3.2	142±6.1	274±9.1	279±9.1
Solvent control	DMSO	133±4.9	139±6.6	35±2.1	39±1.0	133±4.5	141±6.1	277±8.2	278±9.5
ICR-191	1.0	1276±309.2	/	/	/	/	/	/	/
Daunorubicin	6.0	/	/	1243±256.4	/	/	/	/	/
NaN3	1.5	/	/	/	/	1493±201.2	/	/	/
Mitomycin C	0.5	/	/	/	/	/	/	1253±214.6	/
2-AF	10	/	1159±93.0	/	1256±154.0	/	1327±239.3	/	/
1,8-Dihydroxyanthraquinone	50	/	/	/	/	/	/	/	1217±189.8

4.4 Mouse bone marrow cell micronucleus test:

The experimental results are shown in Table 5. The PCE/RBC ratio in each group was not less than 20% of the negative control group, suggesting no bone marrow suppression. According to statistics, the micronucleus rate in each dose group was compared with the negative control group (Poisson distribution), and the differences were not significant ($p > 0.05$).

Table 5. Results of the sample in the mouse bone marrow cell micronucleus test

Sex	Group	Number of mice	Number of PCEs Examined	Number of PCEs with Micronuclei	Micronucleus Rate ‰	p	PCE/RBC
Female	Negative Control	5	5000	6	1. 20±0. 84	/	0. 61±0. 07
	Low Dose	5	5000	6	1. 20±1. 30	0. 554	0. 60±0. 07
	Medium Dose	5	5000	5	1. 00±1. 00	0. 715	0. 59±0. 06
	High Dose	5	5000	7	1. 40±1. 14	0. 394	0. 61±0. 02
	Positive Control	5	5000	117	23. 40±2. 88	<0. 001	0. 53±0. 03
Male	Negative Control	5	5000	6	1. 20±1. 30	/	0. 53±0. 05
	Low Dose	5	5000	6	1. 20±0. 84	0. 554	0. 55±0. 02
	Medium Dose	5	5000	6	1. 20±1. 10	0. 554	0. 55±0. 04
	High Dose	5	5000	7	1. 40±0. 89	0. 394	0. 56±0. 06
	Positive Control	5	5000	138	27. 60±3. 85	<0. 001	0. 51±0. 04

4.5 Mouse sperm abnormality test:

The experimental results are shown in Table 6. The sperm abnormality rate in each dose group was compared with the negative control group (rank sum test), and the differences were not significant (p > 0.05).

Table 6. Results of the sample in the mouse sperm abnormality test

Group	Number of mice	Number of Sperm Examined	Number of Abnormal Sperm						Subtotal (n)	Berration rate %
			Hookless	Fat Head	Banana-Shaped	Double Head/ Tail	Folded	Amorphous		
Negative Control	5	5000	44	0	2	0	3	58	107	2. 14±0. 57
Low Dose	5	5000	54	1	2	0	2	57	116	2. 32±0. 52
Medium Dose	5	5000	54	0	3	0	2	50	109	2. 18±0. 54
High Dose	5	5000	55	0	3	0	2	55	115	2. 30±0. 42
Positive Control	5	5000	107	6	15	2	42	106	278	5. 56±0. 94#

Compared with the negative control group # $p<0.05$

5 Summary:

The acute oral maximum tolerated dose of Sanxan in both male and female rats and mice was greater than 15 g/kg body weight; the Ames test, mouse bone marrow cell micronucleus test, and mouse sperm abnormality test results were all negative. This indicates that under the conditions of this experiment, the sample did not show mutagenicity.

The remainder is blank.

The test sample of Sanxan is an off-white powder, batch number 140421, stored in a cool and dry place. According to GB15193.14-2003, a teratogenicity test was conducted on this sample.
In this experiment, three dose groups were set, namely low, medium, and high doses of 1.3, 2.6, and 5.2 g/kg body weight, equivalent to 60 times, 120 times, and 240 times the recommended human intake. The experimental results showed that no obvious maternal toxicity, embryotoxicity, or teratogenicity was observed in any indicator of each dose group.
(Test methods and results are shown in the attachment)
Teratogenicity test (test methods and results)
1 Materials and methods
1.1 Sample and treatment: The test sample of Sanxan is an off-white powder, batch number 140421, stored in a cool and dry place. The recommended human intake of this product is 1.3 g/60 kg/day.
1.2 Experimental animals and testing conditions: SD rats used in the experiment were provided by Shanghai Slac Laboratory Animal Co., Ltd., production license number SCXK (Hu) 2012-0002, clean grade, 80 female rats and 30 male rats. The experimental animals were acclimated in the animal room environment for 10 days before testing. The body weight of nulliparous female rats was 230-320 g, and that of male rats was 320-360 g. Laboratory animal feed was provided by Zhejiang Laboratory Animal Center, implementing standard GB14924.1-2001. Testing environmental conditions: Laboratory Animal Use License No. SYXK (Zhe) 2013-0190, barrier environment, temperature range 20-23°C, relative humidity range 50-70%.
1.3 Methods
1.3.1 Dose design: Three dose groups and one negative control group (basic feed) were set in the experiment. The low, medium, and high doses were 1.3, 2.6, and 5.2 g/kg, respectively, equivalent to 60 times, 120 times, and 240 times the recommended human intake (consistent with the 90-day feeding test). For each dose group in the experiment, the sample was converted according to 8% of body weight and uniformly mixed into the basic feed, and administered by feeding. Mixing method for the low, medium, and high dose groups: 813, 1625, and 3250 g of the test sample were respectively taken and added with basic feed to 50 kg, and fully mixed. The test substance in the low, medium, and high dose groups accounted for 1.63%, 3.25%, and 6.5% of the feed, respectively. Based on the control feed protein content of 20%, casein was added to the high-dose group feed to adjust the protein content.
1.3.2 Experimental method: SD female and male rats were caged overnight at a 1:1 ratio. Vaginal plugs were observed every morning. If a vaginal plug was found, the rat was considered to have mated, and that day was designated as "day zero" of pregnancy. The detected "pregnant rats" were randomly divided into each group, weighed, and numbered. From day 7 to 16 of pregnancy, the test substance was administered by oral gavage every day. Body weight was measured on days 0, 7, 12, 16, and 20 of pregnancy. The administered dose was adjusted according to body weight changes. On day 20, the rats were sacrificed by cervical dislocation. The uterus was removed and weighed, and the embryos, number of implantations, early resorbed fetuses, late dead fetuses, and number of live fetuses, as well as the external morphology of fetal rats, were recorded and examined. The body weight, body length, and tail length of fetal rats were measured.
Visceral examination: The viscera mainly examined included the brain, heart, lungs, liver, stomach, intestines, and kidneys. One-half of the fetal rats in each litter were placed in Bouin's solution. After fixation for two weeks, the fixative was first rinsed off with tap water. The fetal rats were placed supine on a paraffin board, the limbs and tail were cut off, and a surgical blade was used to make serial transverse or longitudinal sections from the head to the tail. The size, shape, and relative position of organs were observed according to sections at different sites.
Skeletal examination: The skeleton mainly examined included the skull, spine, sternum, ribs, and limb bones. One-half of the live fetuses in each litter were placed in 95% ethanol for fixation for 2-3 weeks. The fetal rats were removed, rinsed with running water for several minutes, and then placed in 2 g/100 mL potassium hydroxide solution for 8-72 h. After clearing, they were placed in alizarin red-S application solution for staining for 6-48 h until the bones were stained red, and then placed in clearing solution until the bones were stained red and the soft tissues were basically decolorized. Then, an overall examination was performed under a stereomicroscope, followed by step-by-step examination of the skeleton.
1.3.3 Result analysis: SPSS11.5 statistical analysis software. First, the homogeneity test of variance was performed on the data. If the variance was homogeneous, one-way ANOVA was used for overall comparison. If there was a difference, pairwise comparison of means with the control group was then performed. If the variance was not homogeneous, the rank sum test was used instead. Various rates were statistically analyzed using the x^2 test.
2 Results
2.1 General observation: During the experiment, no abnormal symptoms or signs were observed in pregnant rats of each group, and there was no death.

2.2 Effect on body weight of pregnant rats

The results are shown in Table 1. There was no significant difference in body weight on days 0, 7, 12, 16, and 20 of gestation, total weight gain, and comparison with the control group among pregnant rats in the negative control group and each dose group (ANOVA, P > 0.05).

Table 1. Effect of Sanxan on body weight of pregnant rats ($\bar{X} \pm SD$)

Group	Number of mice	Gross weight gain	Gestation days 0 d	7 d	12 d	16 d	20 d
Negative Control	12	176.2±21.7	268.1±15.3	307.6±17.4	337.3±17.8	365.0±19.6	444.3±28.2
Low Dose	12	166.2±33.4	272.9±20.2	312.0±21.4	342.9±25.5	371.5±31.1	439.1±41.0
Medium Dose	12	182.7±15.0	281.3±16.7	321.7±18.2	346.6±20.8	371.5±24.8	463.9±23.5
High Dose	12	182.5±35.7	274.9±17.4	320.7±28.7	340.8±37.7	380.2±45.3	457.4±45.8
F		1.074	1.315	1.311	1.155(H)	0.509	1.417
P		0.369	0.280	0.281	0.764	0.678	0.249

2.3 Effect on rat fetal rats

The results are shown in Table 2. The pregnancy rates of the negative control group and the low, medium, and high dose groups were 86.7%, 93.3%, 93.3%, and 86.7%, respectively. The numbers of resorbed fetuses were 7, 6, 7, and 10, respectively, and the numbers of dead fetuses were all 0.

Table 2. Effect of Sanxan on rat fetal rats (Part 1)

Group	Fertilized rat (n)	Pregnant rats (n)	Pregnancy rate (%)	Resorbed Fetuses (n)	Dead Fetuses (n)
Negative Control	15	13	86.7	7	0
Low Dose	15	14	93.3	6	0
Medium Dose	15	14	93.3	7	0
High Dose	15	13	86.7	10	0

The results are shown in Table 3. The raw data of each group did not meet the requirement of homogeneity of variance (P < 0.05). There was no significant difference in uterine weight including fetuses, average number of fetuses, average fetal body weight, and average fetal body length between the low, medium, and high dose groups and the negative control group (ANOVA, P > 0.05).

Table 3. Effect of Sanxan on rat fetal rats (Part 2)

Group	Number of fetuses (n)	Mean Number of Fetuses(n)	Uterine Weight with Fetuses (g)	Mean Fetal Weight (g)	Mean Fetal Body Length (cm)
Negative Control	193	14.8±2.3	98.2±15.9	4.26±0.61	3.85±0.19
Low Dose	208	14.9±4.3	98.2±25.9	4.35±0.68	3.86±0.24
Medium Dose	225	16.1±2.1	103.6±12.9	4.18±0.48	3.80±0.15
High Dose	206	15.9±1.2	104.4±14.9	4.31±0.67	3.82±0.19
H		0.742	0.458	0.181	0.223
P		0.532	0.713	0.909	0.880

2.4 Effect on skeletal development of fetal rats

The results are shown in Table 4. There was no significant difference in the number of sternal segments (including 6, 5, and 4 segments) and the number of the fifth segment appearing as a point between the low, medium, and high dose groups and the negative control group (x^2 test, $P > 0.05$). There was no obvious difference in the number of ribs (13 pairs of ribs and 12 pairs of ribs) between each experimental group and the negative control group. The numbers of fetal rats with incomplete ossification of the parietal bone were 1, 0, 1, and 0, respectively.

Table 4. Effect of Sanxan on skeletal development of fetal rats

Group	Number of Cases (n)	Ribs		Sternal Bones				Incomplete Ossification of the Parietal Bone (n)
		13 pairs	12 pairs	6 Piece (%)	5 Piece (%)	4 Piece (%)	The Fifth is Dot-Like (%)	
Negative Control	99	99	0	95(96.0)	4(4.0)	0(0)	5(5.1)	1
Low Dose	108	107	1	102(94.4)	6(5.6)	0(0)	8(7.4)	0
Medium Dose	116	115	1	106(91.4)	7(6.0)	3(2.6)	5(4.3)	1
High Dose	106	105	1	96(90.6)	7(6.6)	3(2.8)	8(7.5)	0
x^2				3.114	0.700		1.557	
P				0.429	0.429		0.429	

2.5 Effect on viscera of fetal rats

The results are shown in Table 5. Individual fetuses with hydronephrosis and dilation of the renal pelvis were found in the negative control group and the low, medium, and high dose groups, with fetal numbers of 2, 2, 1, and 1, respectively. No abnormalities of the brain, heart, lungs, liver, stomach, intestines, testis (uterus), or bladder were found in the examined fetal rats of each group.

Table 5. Effect of Sanxan on viscera of fetal rats

Group	Number of Fetuses Examined (n)	Number of Fetuses with No Abnormalities (n) (%)	Number of Fetuses with Abnormalities (Hydronephrosis and Renal Pelvis Dilation) (n) (%)
Negative Control	94	92 (97.9)	2
Low Dose	100	98 (98.0)	2
Medium Dose	109	108(99.1)	1
High Dose	100	99(99.0)	1

3.Summary

In this experiment, no obvious maternal toxicity, embryotoxicity, or teratogenicity was observed in any indicator of each dose group.

The remainder is blank.

The test sample of Sanxan is an off-white powder. The submitting party provided a maximum recommended human dietary intake of 1.3 g/person/day. Three dose groups were set in the experiment, equivalent to 60 times, 120 times, and 240 times the recommended human intake, respectively. The experimental results showed that no obvious toxic reaction was observed in any indicator of each dose group in the 90-day feeding study of Sanxan, and the maximum no-observed-adverse-effect level (NOAEL) was 5.2 g/kg body weight.

Report on the 90-Day Feeding Study of Sanxan in Rats

1 Materials and methods

1.1 Sample: The recommended human intake of Sanxan is 1.3 g/60 kg·d, production date 2014.04.21, batch number 1404021. The experiment used the method of mixing into feed for feeding.

1.2 Experimental animals and testing conditions:

Clean-grade SD rats, body weight 60–80 g (Shanghai Slac Laboratory Animal Co., Ltd., production license number SCXK (Hu) 2012-0002).

Feed was provided by Zhejiang Laboratory Animal Center (implementing standard GB14924-2001).

Barrier environment (use license number SYXK (Zhe) 2013-0190), temperature 20–23°C, relative humidity 50–70%. The experimental animals were acclimated in the animal room environment for 3 days before testing.

1.3 Methods

80 SD rats were randomly divided into 4 groups, 20 in each group, half male and half female. The maximum gavage concentration of the sample (deionized water) was 25%. The experiment used the method of mixing into feed for feeding. In the preliminary test, when the sample mixing amount was higher than 6.5%, the feed hardness was too high, affecting the food intake of experimental rats. Therefore, three dose groups of sample 1.3, 2.6, and 5.2 g/kg (equivalent to 60 times, 120 times, and 240 times the recommended human intake) and a negative control group (basic feed) were set in the experiment. For each dose group in the experiment, the sample was converted according to 8% of body weight and uniformly mixed into the basic feed, and administered by feeding. Mixing method for the low, medium, and high dose groups: 813, 1625, and 3250 g of sample were respectively taken and added with basic feed to 50 kg, and fully mixed. The test substance in the low, medium, and high dose groups accounted for 1.63%, 3.25%, and 6.5% of the feed, respectively (the high-dose group was adjusted for protein content with casein). Administration continued for 90 days.

The experimental rats were housed individually with free access to food and water. Continuous observation was conducted for 90 days. Body weight was recorded once a week and food intake twice a week, and food utilization rate was calculated. Blood was collected from the retro-orbital venous plexus during the experimental period (within the seventh week). At the end of the experiment (end of the 13th week), the final body weight was measured, and the animals were fasted overnight. The next day, fasting body weight was measured, and blood was collected from the jugular vein. Hematological indicators were measured with a Sysmex 1800i automatic blood cell analyzer. Blood biochemical indicators were measured with a TBA-40FR automatic biochemical analyzer. After blood collection at the end of the experiment, all animals were subjected to gross anatomy. The liver, kidney, spleen, and testis were taken and weighed, and the organ/body weight ratio was calculated. The liver, kidney, spleen, stomach, intestine, and testis (ovary) were subjected to histopathological examination (fixed in 10% neutral formalin solution, paraffin sections, H-E staining, light microscopy).

1.4 Result analysis: SPSS11.5 statistical analysis. First, the homogeneity test of variance was performed on the data. If the variance was homogeneous, one-way ANOVA was used for overall comparison. If there was a difference, pairwise comparison of means with the control group was then performed. If the variance was not homogeneous, the rank sum test was used instead.

2 Results

2.1 General observation

In the early stage of the experiment, some rats in the medium-dose and high-dose groups showed sticky feces with a small amount of mucous material. This gradually disappeared by about 3–4 weeks, and the fecal characteristics of all rats returned to normal. During the rest of the experimental period, no other abnormal symptoms or signs were observed in rats of each group. No rats died during the experiment.

2.2 Effect on body weight of rats

The results are shown in Tables 1 and 2.

There was no significant difference in body weight between each dose group and the negative control group at each time point of the experiment ($p > 0.05$).

Table 5. Weekly food utilization rate of rats in the 90-day feeding study of the sample (Part 1) ($\bar{x} \pm s$)

Sex	Group	Number of mice	weekly food utilization rate %			
			Week 1	Week 2	Week 3	Week 4
Female	Negative Control	10	43.1±3.4	28.5±7.1	18.0±5.5	12.3±6.2
	Low Dose	10	44.5±3.6	28.6±4.2	14.8±3.7	12.6±5.6
	Medium Dose	10	45.4±4.5	25.2±4.3	13.4±3.7	10.2±2.2
	High Dose	10	40.5±5.1	28.1±6.5	15.9±3.2	8.8±3.0
	F		2.673	0.812	2.297	1.520
	p		0.062	0.496	0.094	0.226
Male	Negative Control	10	49.8±2.5	38.2±2.6	29.7±6.3	23.4±2.1
	Low Dose	10	49.7±4.6	35.6±2.0	26.8±6.8	21.3±3.1
	Medium Dose	10	46.9±2.0	35.9±3.0	24.5±5.2	21.2±5.2
	High Dose	10	46.0±5.0	34.3±6.7	23.5±4.2	20.5±2.7
	F		2.718	7.584(H)	2.304	1.345
	p		0.059	0.055	0.093	0.275

(H) rank-sum test

Table 6. Weekly food utilization rate of rats in the 90-day feeding study of the sample (Part 2) ($\bar{x} \pm s$)

Sex	Group	Number of mice	weekly food utilization rate %				
			Week 5-6	Week 7-8	Week 9-10	Week 11-12	Week 13
Female	Negative Control	10	8.5±2.4	6.5±2.3	4.7±2.2	2.2±0.7	3.1±2.4
	Low Dose	10	8.7±2.1	6.8±2.7	5.9±2.5	3.8±1.1	3.8±2.0
	Medium Dose	10	9.0±1.9	5.0±2.7	5.9±1.5	3.2±1.6	5.8±3.8
	High Dose	10	9.7±2.5	4.8±1.8	6.8±2.1	3.3±1.5	3.5±2.8
	F		0.500	1.799	1.723	2.815	1.889
	p		0.685	0.165	0.180	0.053	0.149
Male	Negative Control	10	13.1±2.5	8.1±1.9	7.2±3.4	6.4±2.1	4.2±3.1
	Low Dose	10	14.1±3.0	8.4±3.0	7.0±1.7	7.2±1.4	4.4±2.3
	Medium Dose	10	12.2±4.4	7.8±3.3	8.9±2.6	8.0±1.3	6.6±1.5
	High Dose	10	15.9±3.4	6.2±2.4	8.3±1.6	7.8±1.9	3.9±2.5
	F		2.255	1.373	1.381	1.670	2.606
	p		0.099	0.266	0.264	0.191	0.067

2.4 Effect on total weight gain, total food intake, and total food utilization rate of rats

The results are shown in Tables 7–9.

The food utilization rate of female rats in the low-dose group was higher than that of the control group, and the difference was significant ($p < 0.05$).

There was no significant difference in total body weight gain, total food intake, and total food utilization rate between each dose group and the negative control group in the first half of the experiment (first six weeks), the second half of the experiment (last seven weeks), and the overall period ($p > 0.05$).

Table 9. Total food utilization rate of rats in the 90-day feeding study of the sample $(\bar{x} \pm s)$

Sex	Group	Number of mice (n)	Body Weight Gain g	Total Food Intake g	Total Food Utilization Rate %
Female	Negative Control	10	223.6±24.2	2192.4±195.4	10.2±0.4
	Low Dose	10	231.3±16.7	2127.2±162.5	10.9±0.5#
	Medium Dose	10	205.4±25.7	2026.3±164.5	10.1±0.7
	High Dose	10	216.7±24.7	2149.0±167.5	10.1±0.7
	F		2.267	1.655	4.092
	p		0.097	0.194	0.013
Male	Negative Control	10	465.3±28.2	3236.1±145.4	14.4±0.5
	Low Dose	10	430.7±51.3	3039.8±207.4	14.1±0.9
	Medium Dose	10	442.7±23.9	3136.5±132.9	14.1±0.5
	High Dose	10	435.8±45.8	3179.1±227.3	13.7±1.1
	F		1.528	2.055	3.552(H)
	p		0.224	0.123	0.314

(H) rank-sum test

#Compared with the negative control group $(p < 0.05)$

2.5 Effect on hematology of rats

The results are shown in Table 10. The values were all within the historical control range of this laboratory. There was no significant difference in hemoglobin, red blood cell count, and white blood cell count between each dose group and the negative control group during the mid-term and end of the experiment (p > 0.05).

Table 10. Hemoglobin, red blood cell count, and white blood cell count of rats in the 90-day feeding study of the sample $(\bar{x} \pm s)$

Sex	Group	Number of mice (n)	Mid-Term			Final-Term		
			Hemoglobin g/L	Red Blood $\times 10^{12}$/L	White Blood Cell $\times 10^{9}$/L	Hemoglobin g/L	Red Blood $\times 10^{12}$/L	White Blood Cell $\times 10^{9}$/L
Female	Negative Control	10	174.2±7.6	9.3±0.4	8.9±2.4	158.4±6.0	8.7±0.3	7.1±1.6
	Low Dose	10	167.6±4.9	9.0±0.4	7.8±2.8	153.2±4.7	8.4±0.3	5.9±1.4
	Medium Dose	10	164.2±18.2	8.9±1.1	8.6±2.2	157.5±4.3	8.7±0.3	7.8±1.5
	High Dose	10	168.0±11.9	9.1±0.6	9.1±3.1	158.6±5.1	8.7±0.2	7.2±1.7
	F		1.250	0.603	0.469	2.468	2.824	2.734
	p		0.306	0.617	0.706	0.078	0.052	0.058
Male	Negative Control	10	167.3±21.6	8.7±1.2	11.2±5.5	160.3±5.5	8.8±0.2	10.9±3.2
	Low Dose	10	171.2±21.6	9.2±1.2	9.4±3.4	158.5±4.3	8.8±0.3	11.2±3.9
	Medium Dose	10	184.2±7.9	10.0±0.7	9.9±5.0	156.2±5.7	8.7±0.3	8.7±1.6
	High Dose	10	179.0±15.6	9.4±0.7	8.9±3.3	162.4±6.9	8.9±0.3	8.4±1.5
	F		1.870	2.823	0.482	2.163	1.008	4.594(H)
	p		0.152	0.052	0.697	0.109	0.400	0.204

(H) rank-sum test

2.6 Effect on WBC of rats

The results are shown in Tables 11–12. The values were all within the historical control range of this laboratory. There was no significant difference in the classification of white blood cells (lymphocytes, monocytes, and granulocytes) between each dose group and the negative control group during the mid-term and end of the experiment (p > 0.05).

Table 11. White blood cell classification of rats during the mid-term of the 90-day feeding study of the sample ($\bar{x} \pm s$)

Sex	Group	Number of mice (n)	Lymphocytes %	Monocytes %	Neutrophils %	Eosinophils %	Basophils %
Female	Negative Control	10	64.78±9.93	2.78±0.68	29.83±9.65	2.56±1.77	0~0.2
	Low Dose	10	65.40±7.07	2.68±0.92	29.51±7.57	2.31±1.04	0~0.4
	Medium Dose	10	63.07±6.22	3.39±0.85	29.85±6.51	3.62±1.20	0~0.4
	High Dose	10	65.56±5.44	2.70±0.46	28.43±4.88	3.30±1.26	0~0.1
	F		0.239	2.039	0.083	2.090	
	p		0.868	0.126	0.969	0.119	
Male	Negative Control	10	61.26±12.86	2.58±0.99	30.93±11.51	5.13±4.78	0~0.4
	Low Dose	10	64.59±8.57	2.71±0.96	29.45±8.31	3.13±1.44	0~0.5
	Medium Dose	10	68.29±3.09	3.56±1.15	25.17±2.77	2.84±1.01	0~0.4
	High Dose	10	61.28±8.84	3.34±0.50	32.11±7.95	3.20±1.52	0~0.2
	F		3.447(H)	2.622	1.350	0.621(H)	
	p		0.328	0.065	0.274	0.892	

(H) rank-sum test

Table 12. White blood cell classification of rats at the end of the 90-day feeding study of the sample ($\bar{x} \pm s$)

Sex	Group	Number of mice (n)	Lymphocytes %	Monocytes %	Neutrophils %	Eosinophils %	Basophils %
Female	Negative Control	10	69.31±2.04	2.33±0.36	25.35±1.96	2.97±1.24	0~0.2
	Low Dose	10	68.23±3.65	2.40±0.61	26.56±3.30	2.77±0.96	0~0.2
	Medium Dose	10	68.36±3.30	2.69±0.84	26.38±1.91	2.55±0.80	0~0.1
	High Dose	10	66.88±3.41	2.62±0.65	27.45±2.31	3.03±1.00	0~0.2
	F		0.999	0.730	1.249	0.461	
	p		0.404	0.541	0.306	0.711	
Male	Negative Control	10	67.63±2.66	3.13±0.73	26.66±2.06	2.49±0.65	0~0.3
	Low Dose	10	66.75±2.15	3.14±0.80	27.51±2.48	2.53±0.81	0~0.3
	Medium Dose	10	66.86±3.46	2.93±0.82	27.85±3.19	2.33±0.46	0~0.1
	High Dose	10	65.37±3.49	3.02±0.55	29.36±3.29	2.17±0.36	0~0.2
	F		0.987	0.185	1.618	0.763	
	p		0.410	0.906	0.202	0.522	

2.7.2 Effect on blood biochemistry of rats at the end of the experiment
The results are shown in Tables 14–15. The values were all within the historical control range of this laboratory. There was no significant difference in serum alanine aminotransferase, aspartate aminotransferase, urea nitrogen, creatinine, cholesterol, triglycerides, blood glucose, total protein, albumin, globulin, and albumin/globulin ratio between each dose group and the negative control group ($p > 0.05$).

Table 14. Blood biochemical indicators of rats at the end of the 90-day feeding study of the sample (Part 1) ($\bar{x} \pm s$)

Sex	Group	Number of mice (n)	Alanine Aminotransferase U/L	Aspartate Aminotransferase U/L	Blood Urea Nitrogen mmol/L	Creatinine umol/L	Triglycerides mmol/L	Cholesterol mmol/L
Female	Negative Control	10	46.9±9.6	242.3±57.3	6.9±1.1	79.1±3.4	0.54±0.12	2.21±0.30
	Low Dose	10	55.7±8.3	306.8±73.0	6.7±0.8	79.8±3.4	0.67±0.31	2.24±0.41
	Medium Dose	10	52.6±8.7	281.9±21.5	6.3±0.4	77.3±3.6	0.56±0.21	2.16±0.36
	High Dose	10	55.9±10.5	309.5±67.8	5.9±0.6	75.5±4.0	0.61±0.23	2.32±0.50
	F		2.040	2.839	7.741(H)	2.833	0.683	0.281
	p		0.126	0.052	0.052	0.052	0.568	0.838
Male	Negative Control	10	53.6±9.6	278.9±55.4	6.0±0.7	67.5±3.7	0.72±0.20	1.81±0.19
	Low Dose	10	57.7±8.2	293.3±47.5	5.6±0.4	68.6±3.9	0.89±0.18	1.81±0.33
	Medium Dose	10	57.5±7.0	294.2±43.9	6.0±0.8	71.7±5.5	0.80±0.21	1.59±0.20
	High Dose	10	62.5±11.4	309.1±64.9	6.5±0.9	68.6±3.3	0.67±0.16	1.69±0.30
	F		1.565	0.531	2.371	1.866	2.819	1.616
	p		0.215	0.664	0.087	0.153	0.053	0.203

(H) rank-sum test

Table 15. Blood biochemical indicators of rats during the mid-term of the 90-day feeding study of the sample (Part 2) ($\bar{x} \pm s$)

Sex	Group	Number of mice (n)	Blood Glucose mmol/L	Total Protein g/L	Albumin g/L	Globulin g/L	Albumin-to-Globulin Ratio
Female	Negative Control	10	5.93±1.29	70.0±3.9	42.2±2.1	27.8±2.1	1.52±0.08
	Low Dose	10	4.91±0.57	69.2±4.5	42.3±2.7	26.9±2.0	1.57±0.07
	Medium Dose	10	5.21±0.60	69.0±2.3	41.6±1.5	27.4±1.5	1.52±0.09
	High Dose	10	5.17±0.58	66.6±3.7	41.0±2.3	25.6±1.9	1.61±0.11
	F		2.847	1.601	0.717	2.645	2.312
	p		0.051	0.206	0.549	0.064	0.093
Male	Negative Control	10	5.52±0.50	61.0±1.3	37.2±1.0	23.9±0.6	1.56±0.05
	Low Dose	10	5.47±0.80	61.6±1.8	37.5±0.6	24.0±1.6	1.57±0.11
	Medium Dose	10	5.58±0.90	62.0±3.2	37.9±1.2	24.1±2.2	1.58±0.11
	High Dose	10	4.73±0.77	61.5±2.6	37.6±1.1	23.9±2.1	1.58±0.13
	F		2.821	0.269	0.797	0.159(H)	0.117
	p		0.053	0.848	0.504	0.984	0.950

(H) rank-sum test

2.8 Effect on organ/body weight ratio of rats. The results are shown in Tables 16–17.
There was no significant difference in organ weight and organ/body weight ratio of liver, spleen, kidney, and testis between each dose group and the negative control group ($p > 0.05$).

Table 16. Organ weight of rats in the 90-day feeding study of the sample ($\bar{x} \pm s$)

Sex	Group	Number of mice (n)	Liver weight g	Kidney weight g	Spleen weight g	Testis weight g
Female	Negative Control	10	7.35±0.80	1.99±0.25	0.54±0.07	/
	Low Dose	10	7.09±0.63	1.93±0.18	0.57±0.09	/
	Medium Dose	10	6.58±0.72	1.77±0.18	0.52±0.07	/
	High Dose	10	6.67±0.58	1.91±0.19	0.52±0.06	/
	F		2.799	2.224	1.249	/
	p		0.054	0.102	0.306	/
Male	Negative Control	10	13.07±1.95	3.69±0.19	0.94±0.16	3.94±0.24
	Low Dose	10	12.06±1.33	3.32±0.35	0.80±0.13	3.86±0.16
	Medium Dose	10	12.01±0.99	3.42±0.33	0.77±0.07	3.79±0.24
	High Dose	10	11.29±1.08	3.35±0.36	0.79±0.17	3.92±0.16
	F		2.782	2.816	2.765	1.098
	p		0.055	0.053	0.056	0.362

Table 17. Effect of the sample on organ/body weight ratio of rats ($\bar{x} \pm s$) (二)

Sex	Group	Number of mice (n)	Fasting Body Weight g	Liver-to-Body Weight Ratio %	Kidney-to-Body Weight Ratio %	Spleen-to-Body Weight Ratio %	Testis-to-Body Weight Ratio %
Female	Negative Control	10	289.1±22.6	2.54±0.15	0.69±0.05	0.19±0.02	/
	Low Dose	10	284.7±18.7	2.50±0.24	0.68±0.05	0.20±0.03	/
	Medium Dose	10	264.6±22.3	2.48±0.09	0.67±0.03	0.19±0.02	/
	High Dose	10	271.0±23.2	2.46±0.10	0.71±0.05	0.20±0.03	/
	F		2.779	2.679(H)	1.205	0.419	/
	p		0.055	0.444	0.322	0.741	/
Male	Negative Control	10	512.8±35.3	2.54±0.27	0.72±0.03	0.18±0.03	0.77±0.08
	Low Dose	10	475.4±45.3	2.54±0.16	0.70±0.04	0.17±0.03	0.82±0.08
	Medium Dose	10	482.0±22.1	2.49±0.14	0.71±0.05	0.16±0.02	0.79±0.06
	High Dose	10	468.3±42.7	2.41±0.09	0.72±0.05	0.17±0.03	0.84±0.08
	F		2.736	1.156	0.442	0.874	1.753
	p		0.058	0.340	0.725	0.463	0.174

(H) rank-sum test

2.9 Histopathological examination of rats

In the low, medium, and high dose groups and the negative control group, there were 20 SD rats in each group, half male and half female. At the end of the experiment, all experimental rats were subjected to gross examination, and a total of 80 rats were dissected. No obvious abnormalities were found in gross anatomy. Microscopic examination found:

Liver: The liver capsule was intact, the histological structure was normal, the morphology of hepatocytes showed no obvious abnormalities, the arrangement of hepatic cell cords was not disordered, and Kupffer cells showed no proliferation or shedding. A small number of rats showed small focal inflammatory cell infiltration in the hepatic lobules, a small number of rats showed a small amount of inflammatory cell infiltration in the portal area, and a small number of rats showed punctate or small focal hepatocellular necrosis with inflammatory cell infiltration. Individual rats showed a tiny granuloma in the liver parenchyma.

Kidney: The kidney capsule was intact, the histological structure was normal, and the glomeruli and renal tubules were evenly distributed; the proximal and distal convoluted tubules and the loop of Henle were closely arranged; the glomeruli showed no filling or atrophy, no inflammatory exudate in the glomerular capsule, no necrosis or shedding of renal tubular epithelium, and no interstitial exudate. The renal pelvic mucosa was intact, with no abnormal changes such as metaplasia. A small number of rats showed granular degeneration of renal tubular epithelial cells, distributed in patches. Individual rats showed inflammatory cell infiltration in the cortical interstitium, distributed in small patches, and focal glomerular calcification, which was mild.

Spleen: The spleen capsule was intact, the histological structure was normal, the splenic corpuscles showed no atrophy, the red pulp and white pulp were visible, the arterial sheath was visible in the white pulp, and scattered lymphocytes and red blood cells were visible in the red pulp. Individual rats showed mild dilation and congestion of the splenic sinuses.

Stomach and intestine: The gastric and intestinal mucosa were intact, with no atrophy, hyperplasia, or necrotic desquamation of epithelial cells, and no hemorrhage or edema in the lamina propria, submucosa, muscular layer, or serosa.

Testis: The histological structure was normal, the seminiferous tubules showed no atrophy, the basement membrane was intact, normally developing spermatogenic cells were visible, and the interstitium was normal.

Ovary: A large number of growing follicles at various stages of development and corpora lutea were visible, and the interstitium showed no abnormalities.

Most of the above-mentioned histological changes were sporadic, and the difference between the negative control group and the high-dose group was not obvious. The occurrence of various histological changes in the experimental groups is shown in the table below.

The remainder of this page is blank.

Table 18. Summary of histopathological observation results

Organs	Pathological Changes (Cases)	Negative Control		High Dose	
		Male n=10	Female n=10	Male n=10	Female n=10
Liver	Mild Congestion of Central Veins in Lobules	0	0	0	0
	Sinusoidal Congestion in the Liver	0	0	0	0
	Granular Degeneration of Cells	0	0	0	0
	Vacuolar Degeneration of Cells	0	0	0	0
	Small Focal Inflammatory Cell Infiltration in Lobules	1	1	1	1
	Inflammatory Cell Infiltration in Portal Areas	2	0	0	0
	Focal Necrosis with Inflammatory Cell Infiltration	0	2	2	0
	Microgranuloma	1	0	0	0
Kidneys	Swelling of Renal Tubular Epithelial Cells	1	0	1	0
	Patchy Inflammatory Cell Infiltration in the Cortical Interstitium	0	0	1	0
	Mild Calcification of Glomeruli	0	0	1	0
	Mild Dilation and Congestion of Cortical Interstitial Blood Vessels	0	0	0	0
	Calcium Salt Deposition in Renal Tubules	0	0	0	0
	Hydropic Degeneration of Papillary Duct Epithelial Cells	0	0	0	0
Spleen	Mild Dilation and Congestion of Sinusoids	0	1	0	0
	Thickening of the Lymphoid Sheath Around Central Arteries	0	0	0	0
	Reactive Hyperplasia of Lymphoid Follicles	0	0	0	0
Gastrointestinal Tract	Congestion of Microvessels in the Lamina Propria	0	0	0	0
	Congestion of Blood Vessels in the Submucosa	0	0	0	0
	Degeneration and Necrosis of the Mucosal Epithelium	0	0	0	0
	Inflammatory Cell Infiltration in the Mucosal Layer	0	0	0	0
Testes	Reduction in Germ Cells and Spermatogenic Dysfunction	0	0	0	0
	Atrophy of Seminiferous Tubules	0	0	0	0
	Hyperplasia of Interstitial Cells	0	0	0	0
Ovaries	Ovarian Cysts	0	0	0	0
	Ovarian Hemorrhage	0	0	0	0
	Hyperplasia of Interstitial Cells	0	0	0	0

3. Summary

The results of this experiment showed that no obvious toxic reaction was observed in any indicator of the 90-day feeding study of Sanxan in rats, and its maximum no-observed-adverse-effect level (NOAEL) was 5.2 g/kg body weight.

The remainder is blank.
